# Rolling Bearing Fault Monitoring for Sparse Time-Frequency Representation and Feature Detection Strategy

**DOI:** 10.3390/e24121822

**Published:** 2022-12-14

**Authors:** Jiahui Tang, Jimei Wu, Jiajuan Qing, Tuo Kang

**Affiliations:** 1 School of Mechanical and Precision Instrument Engineering, Xi’an University of Technology, Xi’an 710048, China; 2 Faculty of Printing, Packing and Digital Media Engineering, Xi’an University of Technology, Xi’an 710054, China

**Keywords:** proximal gradient descent, RCNN, fault diagnosis, rolling bearing

## Abstract

Data-driven fault diagnosis methods for rotating machinery have developed rapidly with the help of deep learning methods. However, traditional intelligent fault diagnosis methods still have some limitations in fault feature extraction and the latest object detection theory has not been applied in fault diagnosis. To this end, a fault diagnosis method based on a sparse short-term Fourier transform (SSTFT) and object detection theory is developed in this paper. First, a sparse constraint is introduced in time-frequency analysis to improve the time-frequency resolution of the model without cross-term interference and proximal gradient descent (PGD) is adopted to quickly and effectively optimize the model to obtain a high-quality time-frequency representation (TFR). Second, a fault diagnosis model based on a region-based convolutional neural network (RCNN) is built; the model can extract multiple regions that can characterize fault features from the TFR. This process avoids the interference of irrelevant vibration components and improves the interpretability of the fault diagnosis model. Finally, multicategory rolling bearing fault identification is realized. The effectiveness of the proposed method is validated by simulation signals and bearing experiments. The results indicate that the proposed method is more effective than existing methods.

## 1. Introduction

Against the backdrop of high-efficiency modern enterprises, equipment failures cause large economic losses and personal safety risks [1,2,3]. Rolling bearing is one of the important components of rotating equipment and rolling bearing failure can easily lead not only to machinery and equipment producing a large security risk but also to increasing enterprise maintenance costs [4,5]. Therefore, effective health monitoring of rolling bearings in mechanical equipment is a better measure to improve the overall safety of equipment and reduce industry production costs [6].

Vibration signals are collected from running rolling bearings and contain information about the health of the equipment. The fault diagnosis method based on vibration analysis is divided mainly into feature extraction, feature selection and fault identification [7,8]. It has been widely demonstrated in some studies that deep learning approaches can extract valuable fault features from raw data without requiring any preconceived signal processing methods. However, in the context of modern equipment, the appearance of heavy background noise, complexity performance and insufficient vibration data, advanced signal processing techniques are required to extract features from the raw data [9,10]. When analysing the operating conditions of rolling bearings in the time domain or frequency domain, the diagnosis can be affected by nonlinear factors, such as stiffness and clearance to vibration signals and load friction. Time-frequency analysis methods are more effective when handling nonlinear and nonstationary signals because these methods can accurately describe the local time-frequency characteristics of nonstationary signals by revealing the frequency components and their time-varying properties [11,12,13,14]. Hence, time-frequency analysis methods have been widely used in fault diagnosis in the past few years. Li et al. [15] proposed a two-direction two-dimensional linear discriminative analysis (TD-2DLDA), which combine the advantages of the short-time Fourier transform (STFT) and wavelet transform. Cai et al. [16] combined the generalized S transform and singular value decomposition (SVD) for time-frequency analysis of bearing fault vibration signals. Dhamande et al. [17] employed continuous and discrete wavelet transforms of the vibration signal to extract compound fault features in gear systems. Chandra et al. [18] investigated the performance of different time-frequency analysis methods in fault diagnosis of rotor bearing systems. Wang et al. [19] developed a sparse and low-rank decomposition of the TFR method for bearing fault diagnosis. Israel et al. [20] combined local mean decomposition (LMD) and the Wigner–Ville distribution (WVD) to propose an efficient time-frequency analysis method.

The aforementioned traditional time-frequency methods can be roughly divided into two categories: linear time-frequency methods (which are represented by the STFT transform and wavelet transform) and nonlinear time-frequency methods (which are represented by the Wigner–Ville distribution). The method in the latter category can characterize faults in the time-frequency domain but has obvious limitations. The methods in the former category are limited by the Heisenberg uncertainty principle, resulting in poor sparsity and low time-frequency resolution in the analysis results. The latter suffers from cross-term interference, which reduces the accuracy of the TFR.

The development of sparse representation theory allows the above problems to be solved. With the application of sparse representation theory in time-frequency analysis, time-frequency methods based on sparse representation and an STFT have emerged. A time-frequency analysis problem can be transformed into a sparse optimization problem based on the L0 norm constraint, and the solution for this problem can obtain a higher resolution time-frequency representation. Although there is no interference from cross-terms, the optimization process for this model is a nonconvex optimization problem, which is a nondeterministic polynomial hard (NP-hard) problem to solve. For this purpose, this paper proposes a time-frequency analysis method based on PGD and a SSTFT. First, the convex relaxation technique is applied to relax the nonconvex optimization problem of the L0 norm to an L1 norm minimization problem and then PGD is used to solve the problem.

Some satisfactory results have been acquired by intelligent bearing fault identification algorithms by using TFR as input. Zhang et al. [21] used the scaled exponential linear unit to improve the learning ability of the convolutional model for TFR. Ma et al. [22] proposed a fault diagnosis model based on time-frequency analysis and a deep residual network. Liang et al. [23] utilized parallel convolutional neural networks (CNNs) for TFR learned by a continuous wavelet transform (CWT). Akhenia et al. [24] applied the single image generative adversarial network (SinGAN) to generate additional TFRs as training samples for the classifier. Wang et al. [25] employed convolution deformable atrous convolution to extract bearing fault features in TFR. Udmale et al. [26] used CNNs to learn fault features in Kurtogram TFRs. Wang et al. [27] proposed a fault diagnosis method based on a multitask CNN by taking the time-domain signals, the frequency-domain signals and the TFR as the input of the CNN at the same time.

However, the abovementioned traditional intelligent diagnosis method based on TFR still has two inherent shortcomings:

(a) When the TFR is used as input, the entire TFR is usually used as the learning object of the model and the understanding of the TFR is lacking. Many irrelevant vibration components also establish relationships with labels, reducing model interpretability.

(b) As one of the cutting-edge ideas in image processing, the application of object detection theory in fault diagnosis is rarely addressed.

To this end, the Faster RCNN algorithm is introduced in the traditional intelligent fault diagnosis based on time-frequency analysis in this paper; the algorithm can accurately mark the fault components from the TFR to accurately realize the fault diagnosis of rolling bearings [28]. Faster RCNN, one of the extensions of the RCNN, is one of the state-of-the-art solutions in general object detection [29]. The method can be divided into two main parts. (1) Region proposal network: This network is used mainly to generate a list of region proposals that may contain objects. (2) Object localization and classification network: This is used mainly for classifying a region of an image into objects (and the background) and refining the boundaries of these regions. The proposed method follows a framework similar to Faster RCNN. Here, the fault component is taken as the target to be identified so that the fault category of the original TFR can be accurately identified towards higher recall and accuracy. Compared with the traditional method (which uses the entire TFR as the model mapping object), the proposed method can directly point out the fault components in the TFR, thus improving the interpretability of the intelligent diagnosis method.

Inspired by the expectation of addressing the abovementioned problems, a bearing fault diagnosis method based on PGD-SSTFT and Faster RCNN is proposed in this paper. The PGD-SSTFT algorithm is adopted to obtain a sparse TFR without cross-term interference from the vibration signal and the fault characteristics of rolling bearings can be displayed with high resolution. A fault diagnosis model is then built based on Faster RCNN, one of the representative algorithms in object detection theory and the model is used to learn the fault feature components from the TFR. Finally, the fault components can be accurately marked in the bearing fault samples of unknown categories and the fault type can be identified.

The main contributions of this paper are as follows:

(1) A time-frequency analysis model of the bearing vibration signal is built by using the STFT and sparse constraints are introduced into this model, thereby improving the time-frequency resolution and time-frequency aggregation of the time-frequency analysis method. Additionally, the model is transformed into an easy-to-solve unconstrained problem by using the PGD algorithm.

(2) The object detection method is introduced in fault diagnosis and the fault feature components are more accurately and pertinently labelled from the original TFR, thereby improving the interpretability of the fault diagnosis method.

(3) The effectiveness of the proposed method is validated by using the simulated signal and the actual bearing vibration signal. The results indicate that the TFR of the proposed method is more accurate than that of the traditional method and the fault identification accuracy is higher.

The remaining parts are organized as follows: Section 2 introduces the basic theory of sparse time-frequency analysis methods based on PGD-SSTFT and Section 3 presents a fault diagnosis model based on object detection theory. Section 4 establishes the end-to-end fault diagnosis model based on the proposed method. Section 5 conducts experiments to test the effectiveness of the proposed method. The conclusions are summarized in Section 6.

## 2. Sparse Time-Frequency Decomposition of Proximal Gradient Descent

### 2.1. Sparse Time-Frequency Decomposition

The short-time Fourier transform is a joint time-frequency analysis method for time-varying nonstationary signals and is one of the most basic tools for time-frequency analysis of actual signals. The basic idea of the STFT can be summarized as follows: A fixed-length window function is utilized to intercept the time-domain signal and the Fourier transform is adopted to process the resulting truncated signal, thus obtaining a local Fourier spectrum of the short period. This process is completed until the window function spectrum is translated over the complete time range and a set of local spectra is obtained for each period. Hence, the STFT is a two-dimensional function of time and frequency and can be calculated as

(1) $$\begin{array}{c} \begin{array}{c} F\left\{x(t)\right\}\left(t,w\right)=\int_{-\infty }^{+\infty }x(\tau )w\left(\tau -t\right)\exp\left(-jw\tau \right)d\tau \end{array} \end{array}$$

where w(τ−t) is a sliding window function.

x(n) represents a discrete signal with period *n* and the form of the signal’s discrete STFT can be defined as

(2) $$\begin{array}{c} \begin{array}{c} F\left\{x(n)\right\}\left(m,l\right)=\sum_{n=0}^{N-1}x\left(n\right)w\left(n-m\right)W_{N}^{nl} \end{array} \end{array}$$

where WN=exp(−j2π−j2πNN).

According to the inverse Fourier transform, the original signal is windowed at time *m*. $x_{m}\left(n\right)$ can be obtained as

(3) $$\begin{array}{c} \begin{array}{c} x_{m}\left(n\right)=x\left(n\right)w\left(n-m\right)=\frac{1}{N}\sum_{l=0}^{N-1}F\left\{x(n)\right\}\left(m,l\right)W_{N}^{-nl} \end{array} \end{array}$$

The matrix formula for this transformation can be expressed as

(4) $$\begin{array}{c} \begin{array}{c} x_{m}=W_{N}X_{m} \end{array} \end{array}$$

where $x_{m}$ is the windowed signal at time *m*, $X_{m}$ is the Fourier spectrum at time *m*, $W_{N}$ is an inverse Fourier matrix.

The short-term signal obtained after introducing the window function is

(5) $$\begin{array}{c} \begin{array}{c} y_{m}=G_{m}x_{m}=G_{m}W_{N}X_{m} \end{array} \end{array}$$

where $y_{m}$ is the short-term signal truncated by the window function and $G_{m}$ is the sliding window function at time *m*. $G_{m}W_{N}$ is a partial Fourier matrix and its column vectors are correlated, so $X_{m}$ may not have a unique solution.

The frequency domain of the windowed signal $x_{m}$ at time *m* is sparse. The special spectrum $X_{m}$, inspired by sparse representation theory, may be readily generated by adding the sparse restriction to suit the sparse prior knowledge of the spectrum. The process can be described as

(6) $$\begin{array}{c} \begin{array}{c} {\hat{X}}_{m}=\arg\min_{X_{m}}{∥X_{m}∥}_{0}s.t.y_{m}=G_{m}W_{N}X_{m} \end{array} \end{array}$$

Finding a solution to the L0 norm is very complex; this problem is called an NP-hard problem and this process is prone to noise interference. To address this problem, an alternative method is explored in this paper. It has been proven by some studies [30,31,32,33] that the nonconvex optimization problem of the L0 norm is relaxed to an L1 norm minimization problem that can also obtain a sparse solution. In other words, the L1 norm constraint makes the problem to be solved behave as a convex optimization problem and can thus obtain the optimal solution by using the linear programming method. Therefore, an ideal approximation can be derived by solving the following convex optimization problem.

(7) $$\begin{array}{c} \begin{array}{c} {\hat{X}}_{m}=\arg\min_{X_{m}}{∥X_{m}∥}_{1}s.t.{∥G_{m}W_{N}X_{m}-y_{m}∥}_{2}\leq \varepsilon \end{array} \end{array}$$

where ${∥X_{m}∥}_{1}=\sum_{m}\left|X\right|$ is the L1 norm of the vector X. ${\hat{X}}_{m}$ represents the estimated signal spectrum at time *m*, ε is a bound for noise and interference in the data.

The TFR of the signal ${\hat{X}}_{m}=\left[{\hat{X}}_{0},{\hat{X}}_{1},{\hat{X}}_{2}\cdots {\hat{X}}_{N-1}\right]$ can be obtained by solving the optimization problem of Equation (7) for all m=0,1,2…,N−1 and this process can show the sparse time-frequency analysis model. Considering the signal dimension, the model may be a large-scale optimization problem, which necessitates a fast and efficient algorithm to estimate ${\hat{X}}_{m}$. The details of this algorithm will be discussed in the next subsection.

### 2.2. A Solution Method Based on Proximal Gradient Descent

Since the above model is a set of convex optimization problems with constraints, the constrained problem is converted into an unconstrained problem to facilitate the solution [34,35]:

(8) $$\begin{array}{c} \begin{array}{c} {\hat{X}}_{m}=\arg\underset{X_{m}}{\min\mu }{∥X_{m}∥}_{1}+\frac{1}{2}{∥G_{m}W_{N}X_{m}-y_{m}∥}_{2}^{2} \end{array} \end{array}$$

Let $g\left(X_{m}\right)={∥G_{m}W_{N}X_{m}-y_{m}∥}_{2}^{2}$ and expand it by using a Taylor series at $X_{0}$:

(9) $$\begin{array}{c} \begin{array}{cc} g\left(X\right)\approx g\left(X_{0}\right) & +\nabla g\left(X_{0}\right)\left(X_{m}-X_{0}\right)\\ \; & +\frac{1}{2}\nabla^{2}g\left(X_{0}\right){\left(X_{m}-X_{0}\right)}^{2} \end{array} \end{array}$$

where $\nabla^{2}g\left(X_{0}\right)=\frac{1}{t}$.

(10) $$\begin{array}{c} \begin{array}{cc} {\hat{X}}_{m}= & \arg\underset{X_{m}}{\min\mu }{∥X_{m}∥}_{1}+\frac{1}{2}g\left(X_{0}\right)+\frac{1}{2}\nabla g\left(X_{0}\right)\left(X_{m}-X_{0}\right)+\frac{1}{4t}{\left(X_{m}-X_{0}\right)}^{2} \end{array} \end{array}$$

Equation (10) obtains the maximum value of $X_{m}$ and $g(X_{0})$ has no effect on the result, so this term can be removed and a constant term can be introduced as

(11) $$\begin{array}{c} \begin{array}{c} \begin{array}{c} {\hat{X}}_{m}\approx \arg\underset{X_{m}}{\min\mu }{∥X_{m}∥}_{1}+\frac{1}{4t}\left[{\left(X_{m}-X_{0}\right)}^{2}+2t\nabla g\left(X_{0}\right)\left(X_{m}-X_{0}\right)+(t\nabla g\left(X_{0}\right){)}^{2}\right]\\ =\arg\underset{X_{m}}{\min\mu }{∥X_{m}∥}_{1}+\frac{1}{4t}{\left[X_{m}-X_{0}+t\nabla g\left(X_{0}\right)\right]}^{2} \end{array} \end{array} \end{array}$$

Let $z=X_{0}-t\nabla g\left(X_{0}\right)$; the equation can be rewritten as

(12) $$\begin{array}{c} \begin{array}{c} {\hat{X}}_{m}=\arg\underset{X_{m}}{\min\mu }{∥X_{m}∥}_{1}+\frac{1}{4t}{∥X_{m}-z∥}_{2}^{2} \end{array} \end{array}$$

In the general calculation process, Equation (12) is called the proximal operator, which can be denoted as

(13) $$\begin{array}{c} \begin{array}{c} prox_{t,{\left\|\right\|}_{1}}\left(z\right)=\arg\underset{X_{m}}{\min\mu }{∥X_{m}∥}_{1}+\frac{1}{4t}{∥X_{m}-z∥}_{2}^{2} \end{array} \end{array}$$

In addition, a soft threshold function is introduced:

(14) $$\begin{array}{c} \begin{array}{c} X_{m}=S_{\mu }(z)=\left\{\begin{array}{c} z+\mu z<-\mu \\ 0z<\left|\mu \right|\\ z-\mu z>\mu \end{array}\right. \end{array} \end{array}$$

The analytical solution of Equation (12) can be obtained as

(15) $$\begin{array}{c} \begin{array}{c} {\hat{X}}_{m}=\arg\underset{X_{m}}{\min\mu }t{∥X_{m}∥}_{1}+\frac{1}{4}{∥X_{m}-z∥}_{2}^{2}=S_{\mu t}(z) \end{array} \end{array}$$

According to the proximal gradient descent, the general iterative process for the problem of sparse time-frequency decomposition can be calculated as

(16) $$\begin{array}{c} \begin{array}{c} z^{\left(k\right)}=X_{m}^{\left(k\right)}-t\nabla g\left(X_{m}^{\left(k\right)}\right) \end{array} \end{array}$$

(17) $$\begin{array}{c} \begin{array}{cc} X_{m}^{k+1}=prox_{t,{\left\|\right\|}_{1}}\left(z^{\left(k+1\right)}\right) & =\arg\underset{X_{m}}{\min\mu }t{∥X_{m}∥}_{1}+\frac{1}{4}{∥X_{m}-z^{\left(k\right)}∥}_{2}^{2} \end{array} \end{array}$$

### 2.3. Simulation Analysis

To verify the effectiveness of the proposed proximal gradient time-frequency method, simulated bearing fault signals are used in this section.

The collected signals of bearing vibration in the industry are usually complex, have multiple components and have a low signal-to-noise ratio (SNR). Hence, on the basis of existing research, the faulty rolling bearings vibration signal model proposed in this section can be expressed as [36,37]

(18) $$\begin{array}{c} \begin{array}{c} \left\{\begin{array}{c} x_{inner}\left(t\right)={\sum_{i}A}_{i}h\left(t-iT-\tau_{i}\right)+n\left(t\right)\\ h\left(t\right)=e^{-\gamma t}\cos\left(2\pi f_{n}t+\phi_{\omega }\right) \end{array}\right. \end{array} \end{array}$$

(19) $$\begin{array}{c} \begin{array}{c} A_{inner}=A_{0}\cos2\left(2\pi f_{r}t+\phi_{A}\right)+C_{A} \end{array} \end{array}$$

where Equation (18) is the vibration simulation signal of the bearing fault; A is the amplitude modulation caused by the simulated load change; *T* represents the mean period of fault impact; $n\left(t\right)$ denotes the noise component; τ simulates the relative slip of impacts; $h\left(t\right)$ is the impact pulse function, which can be initially described by a cosine function; $f_{n}$ denotes the resonant frequency; γ denotes the impact decay rate; and these frequencies are associated with the repetitive pulse signal generated by the fault.

Since the inner race rotates with the running rolling bearing, the fault point sometimes appears in the load area, sometimes not in the load area, but the position of the sensor is fixed. Therefore, the fault points are located in different positions, resulting in continuous changes in the magnitude and direction of the generated impulse force. Equation (19) is used to simulate the vibration and impact amplitude of bearing faults, where $f_{r}$ is the rotational frequency. The parameters of the simulated signal are shown in Table 1. The sampling frequency is 12 kHz and the acquisition time is 2 s.

**Figure 1:**
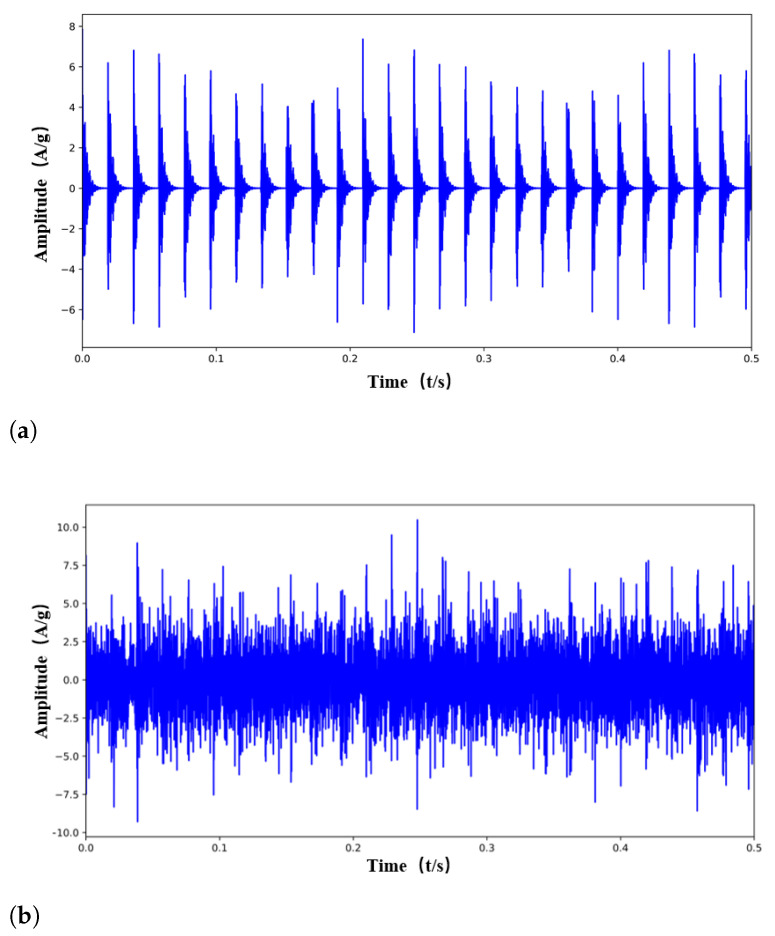
Simulated signal. (**a**) Fault impulses. (**b**) Mixture signal.

Figure 1a illustrates the original time-domain simulated signal and the signal with Gaussian white noise (SNR = −8) that obeys the normal distribution is shown in Figure 1b. From the figure, the periodic characteristics of the simulated signal are partially buried in heavy background noise.

**Figure 2:**
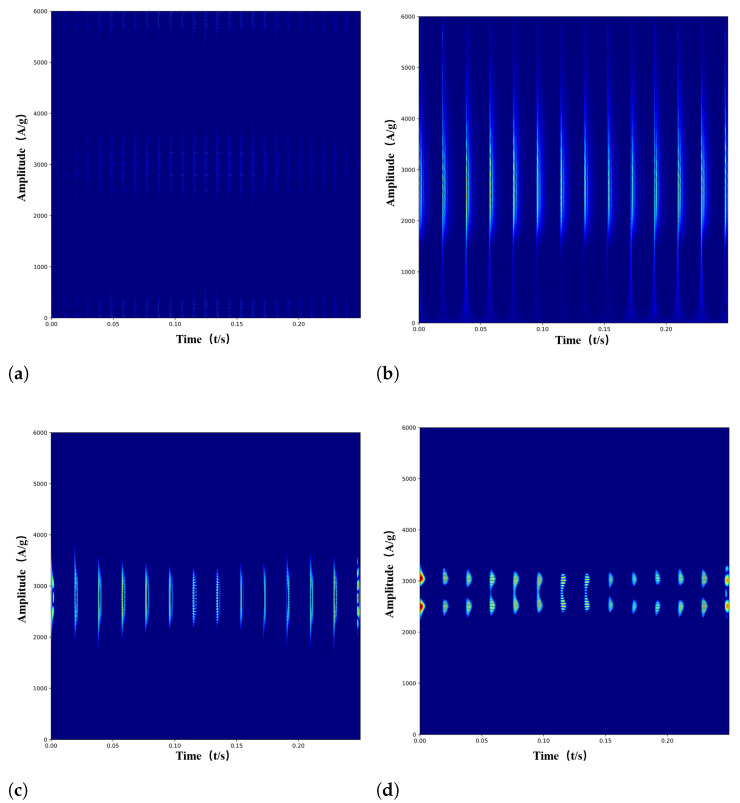
Time-frequency representation. (**a**) WVD. (**b**) CWT. (**c**) SSTFT. (**d**) PGD-SSTFT.

To obtain a high-resolution TFR of bearing fault signals without cross-term interference, the proposed PGD-SSTFT is applied to the simulation signal in this paper. The window function size is 65 (sample points) and ε is 0.1. Additionally, four time-frequency analysis methods (namely, the SSTFT, CWT, WVD and PGD-SSTFT) are used for the simulated signal without noise and the obtained time-frequency image is shown in Figure 2. The figure shows that the time-domain window width of the SSTFT time-frequency image is fixed, the time-frequency resolution is relatively poor, the energy distribution is not concentrated and the time-frequency distribution of the signal cannot be accurately reflected. The WVD is interfered with by the cross-term and cannot accurately reflect the time-frequency characteristics of the signal. The CWT scale does not have a good corresponding relationship with the frequency of the signal, resulting in poor frequency resolution of the TFR and cannot well express the variation in the frequency of each component of the signal with time. However, the PGD-SSTFT method has high resolution, concentrated energy distribution and good time-frequency aggregation, which can accurately reflect the time-frequency energy distribution of the periodic characteristics of the simulated signal.

**Figure 3:**
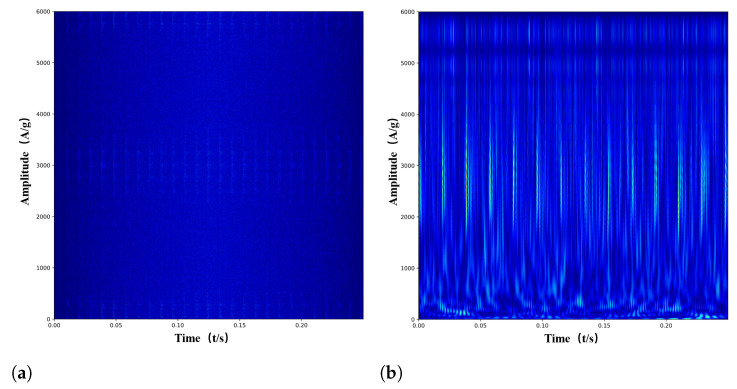

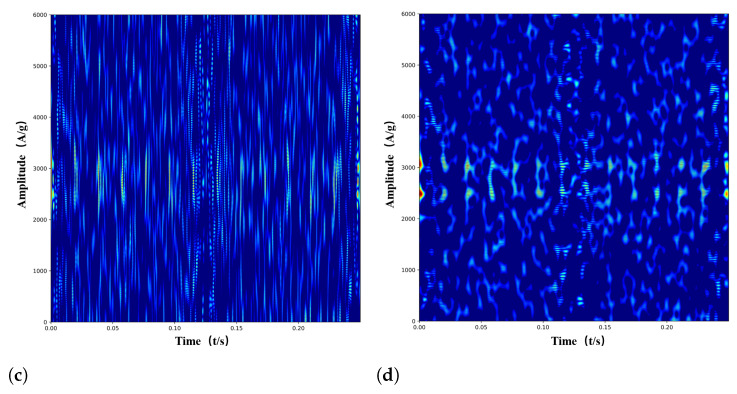
Time-frequency representation (with the SNR of −8 dB). (**a**) WVD. (**b**) CWT. (**c**) SSTFT. (**d**) PGD-SSTFT.

In general, rolling bearings often work in a noisy environment, so the collected signal contains considerable noise. Therefore, a signal containing Gaussian white noise is utilized to test the effectiveness of the proposed method in this paper. Figure 3 shows that the TFRs of the four methods are all disturbed by different levels of noise. However, even in a high-noise environment, the TFR obtained by the proposed method still has a better time-frequency resolution than the competitive models.

**Figure 4:**
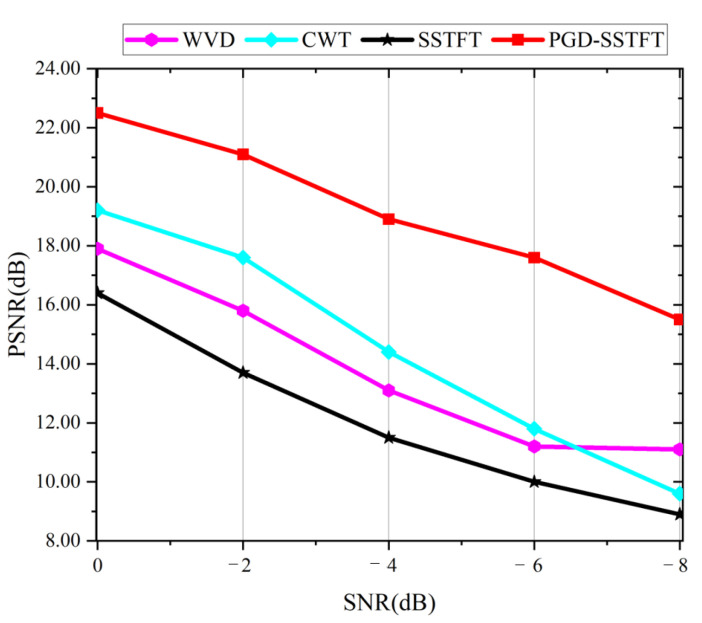
PSNR values obtained by different methods.

To further evaluate the denoising performance of the proposed method, the peak signal-to-noise ratio (PSNR) is selected as the evaluation index. Based on statistics, this method distinguishes the difference between figures by comparing pixel points one by one.

(20) $$\begin{array}{c} \begin{array}{c} PSNR\left(Z,K\right)=10\log_{10}\frac{\max_{Z}^{2}}{\frac{1}{M\times N}\sum_{i=1}^{M}\sum_{j=1}^{N}{\left(Z_{ij}-K_{ij}\right)}^{2}} \end{array} \end{array}$$

where Z is the TFR of the original simulation signal and K is the TFR of the signal with noise. The PSNR value represents the similarity of two images and a larger PSNR value means a higher similarity. Figure 4 shows the PSNR values of four methods under different SNR conditions. As the SNR decreases, the PSNR values of four methods decrease, which proves that noise reduces the effectiveness of each method. It is worth mentioning that the proposed method has the largest PSNR value, which shows the denoising performance of this method is superior to other methods. Even under the interference of −8 db noise, it still achieves a high PSNR value. Thus, the PGD-SSTFT proposed in this paper is an effective TFR method, which has superior performance to traditional time-frequency analysis methods.

## 3. Fault Diagnosis Model Based on Fault Feature Detection

In the second section, the time-frequency decomposition results of PGD-SSTFT are presented. It can be clearly seen from the results that different types of faults have different frequency components, but relying on manual identification of the fault components from the TFR is undoubtedly complex and labor-intensive. Hence, our focus is to automatically identify the fault components in the TFR.

To this end, the Faster RCNN is introduced to directly extract the regions related to the fault components from the original TFR and then the identification model is established to achieve fault diagnosis. Traditional intelligent diagnosis methods usually use the entire TFR corresponding to the fault type to achieve the fault diagnosis, which lacks the understanding of time-frequency information. The difference from the traditional intelligent method is that the proposed method can automatically identify the regions containing the fault frequency from the original TFR and determine the fault type implied in each TFR. This not only considers the location of the fault frequency (the position in the TFR is determined by the horizontal ordinate time and the longitudinal coordinate frequency), but also considers the energy of the fault frequency (determined by the color displayed in the TFR). The proposed method can be divided into the following steps: data preprocessing, fault region proposal, screening algorithm and fault identification.

### 3.1. Data Preprocessing

In this paper, when processing the original time-frequency dataset, software is used to annotate the time-frequency signal and the main fault frequency features in the TFR of different fault types are selected. As shown in the figure, the regions in these boxes include existing features as much as possible and the regions selected in the box can reflect the position and energy of this frequency. This process is realized with existing software. More details on this process can be found in the results.

### 3.2. Fault Region Proposal

**Figure 5:**
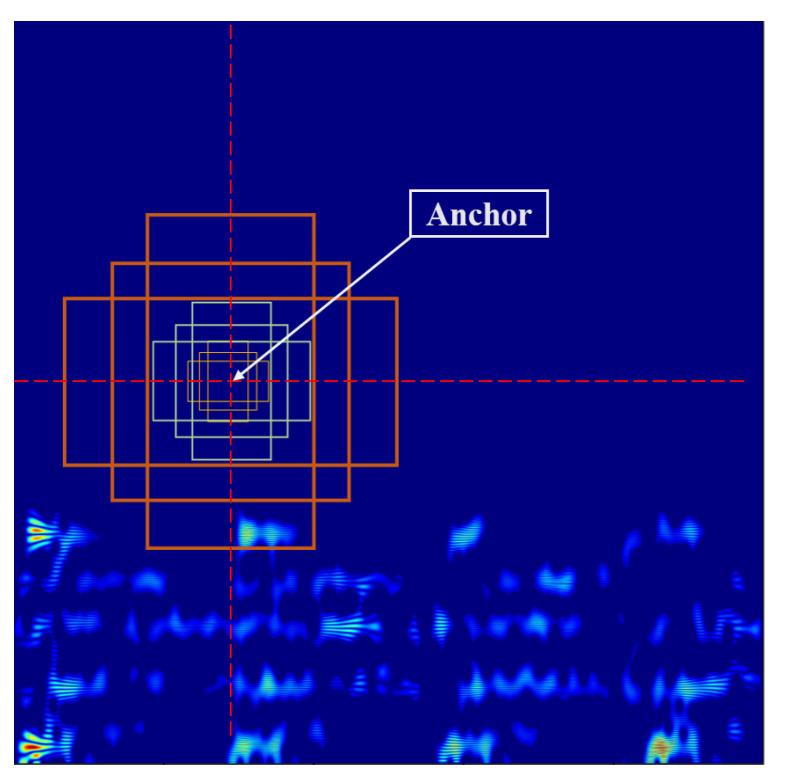
The procedure of fault region proposal.

The input TFR first undergoes feature extraction and then enters the region suggestion step. This process is completed with multiple convolution layer structures and *k* anchors are randomly generated on the input feature map. As illustrated in Figure 5, the image size is H×W and H×W×k anchors will be generated. Then, nine anchor boxes are generated with each anchor as the centre. This process obtains multiple suggested regions on the same input feature map. Therefore, this process outputs the coordinates of these regions and the probability that the box contains different features; this probability is calculated with *Softmax*.

This process can obtain the best region containing fault features but also generates many duplicate anchor regions and can thereby lower the performance of models. Therefore, regions with lower scores are deleted according to the intersection-over-union (IoU) condition. The operation can be summarized as follows: The box with the highest confidence is selected and then the box whose IoU is higher than the threshold is deleted until the best rectangle is selected to achieve the position output of the target. These selected regions are called regions of interest (RoIs).

(21) $$\begin{array}{c} \begin{array}{c} IoU=\frac{area\left(A_{1}\right)\cap area\left(A_{2}\right)}{area\left(A_{1}\right)\cup area\left(A_{2}\right)} \end{array} \end{array}$$

### 3.3. Standardization of Regions of Interest

After feature extraction, the above RoIs are performed on the feature map, which cannot reflect the features in the original TFR. In addition, the above steps result in the suggested regions containing different dimensions, thus making it impossible to complete the subsequent fault classification. For this purpose, a normalization layer similar to spatial pyramid pooling is introduced to map the resulting RoIs to the original TFR and adjust the size for classification. This part consists of multiple convolutional layers and pooling layers.

### 3.4. Fault Identification

After the RoIs are obtained, the fault identification part is entered. The fault identification part completes two main tasks. First, the position regression calculation is performed to calculate the loss of the predicted and the actual regions. Then, the loss will be backpropagated to optimize the parameters.

Suppose the coordinates of the box are $\left(x,y,w,h\right)$, *P* is the proposed box and *G* is the actual box. Then, the goal of model regression is to find a mapping that satisfies $F\left(P_{x},P_{y},P_{w},P_{h}\right)=G_{x},G_{y},G_{w},G_{h}$.

For this purpose, the prediction box is translated and scaled separately.

(22) $$\begin{array}{c} \begin{array}{c} \left\{\begin{array}{c} \overset{\wedge }{G_{x}}=P_{w}d_{x}\left(P\right)+P_{x}\\ \overset{\wedge }{G_{y}}=P_{h}d_{y}\left(P\right)+P_{y} \end{array}\right. \end{array} \end{array}$$

(23) $$\begin{array}{c} \begin{array}{c} \left\{\begin{array}{c} \overset{\wedge }{G_{w}}=P_{w}\exp\left(d_{w}\left(P\right)\right)\\ \overset{\wedge }{G_{h}}=P_{h}\exp\left(d_{h}\left(P\right)\right) \end{array}\right. \end{array} \end{array}$$

The gradient descent algorithm is used to continuously make the predicted box *G* approach the position of the real box. Fault identification completes fault classification, which is realized by adding *Softmax* after the fully connected layer. The final classification part integrates all RoIs in the entire feature map, sorts the probability categories corresponding to these RoIs and outputs the category with the highest probability among all RoIs. During this process, the loss between the predicted box and the ground-truth box is calculated and the loss is backpropagated to optimize the parameters.

### 3.5. The Framework of Proposed Model

**Figure 6:**
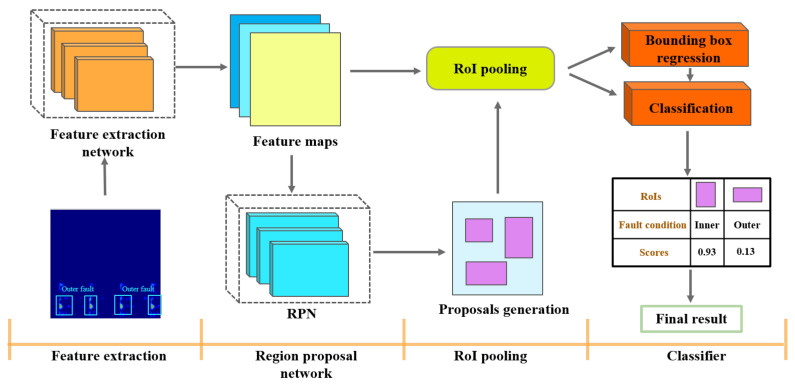
The framework of the proposed model.

In this paper, a fault diagnosis model is built on the basis of Faster RCNN in object detection theory, thus making the neural network more refined with respect to finding fault features from the TFR. As mentioned above, this method can be divided into four main parts (as shown in Figure 6). First, the feature extraction part performs a preliminary extraction of fault features and then the region proposal part extracts proposal regions, which are then processed by the RoI pooling part. The final classification part will integrate the preselected boxes obtained from a TFR to identify the fault type.

## 4. General Procedure of Proposed Fault Diagnosis Method

**Figure 7:**
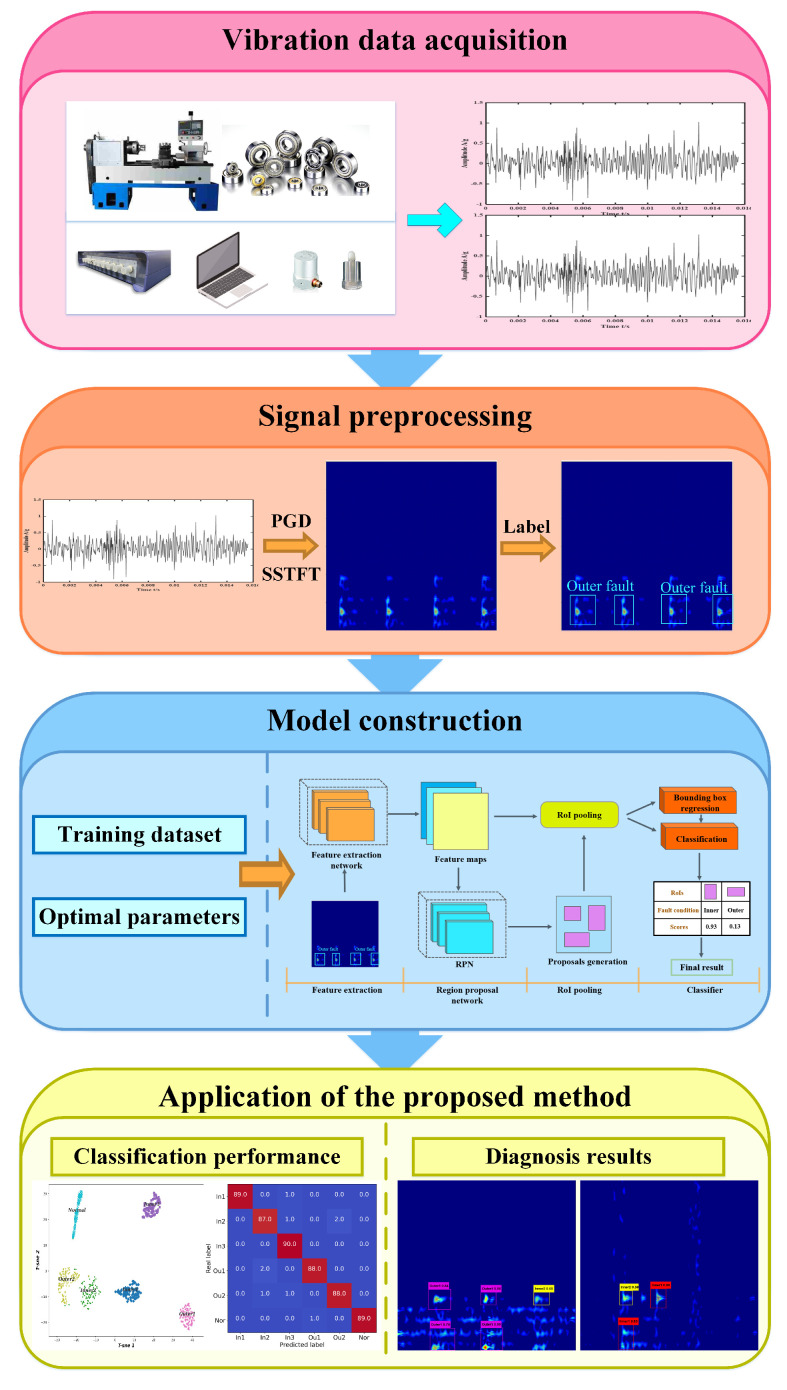
The general diagnosis procedure of the proposed method.

To improve the accuracy and stability of intelligent diagnosis, a rolling bearings fault diagnosis method based on sparse time-frequency decomposition and object detection theory is proposed in this paper. As shown in Figure 7, more details on the framework of the proposed method are shown as follows:

(1) Vibration signals are collected by using acquisition equipment, such as sensors.

(2) The vibration signal is then converted to a TFR by using sparse time-frequency decomposition based on PGD-SSTFT; then, these images are labelled.

(3) A fault diagnosis mode is established based on object detection theory and the optimal related hyperparameters and model structure are determined.

(4) The model is fully trained by using training samples.

(5) The trained model is applied to identify test samples and then the diagnosis results are output and the model performance is evaluated.

## 5. Experimental Studies and Analysis

### 5.1. Data Description

**Figure 8:**
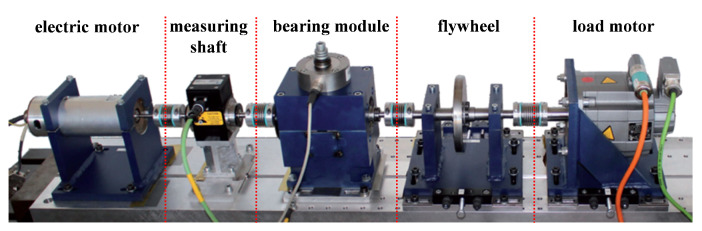
Experimental setup [38].

To verify the effectiveness of the proposed method, data from the chair of Design and Drive Technology, Paderborn University are adopted for testing [38]. The main structure of the test bench, which is shown in Figure 8, consists of a bearing seat and a motor. The bearings used in the test run under the radial load imposed by the spring screw. The settings of the experimental platform are presented in Table 2. To accelerate fatigue damage, this radial force is usually greater than the daily use of bearings but still does not exceed the static load capacity of bearings. The bearings used in the experiment contain two operating states, namely, the normal state and the actual damage fault. The actual damage fault includes fatigue pitting and plastic deformation. More details about the operating state are shown in Table 3. After truncating, the collected signal is processed according to the proposed method in Section 2. These obtained TFRs are labelled as input to the detection model.

### 5.2. Model Selection

In this section, the structure and hyperparameters of the detection model based on cross-experiments on the above datasets are determined and the impact of these parameters on model performance is evaluated. To improve the validity of this study, cross-validation for all hyperparameters that may affect model performance is conducted for multiple average validation and analysis. All parameters include the window function size, the bound of error, batch size, base anchor size and dropout.

The experiments are performed 10 times for each set of parameters for cross-validation and the average results of the cross-experiments are shown in Table 4. Base represents the final selected parameter model and the average accuracy and calculation time are selected as indicators. A blank line in the calculation time indicates that the parameter has little effect on the calculation time. The results reveal that the selection of these parameters affects the performance of the model and unreasonable parameter selection reduces the average accuracy and makes the network performance unstable.

### 5.3. Results and Analysis

To verify the effectiveness of the proposed method, the advanced object detection method Yolo [39] is chosen as the comparison method in the following case study. Three popular fault diagnosis algorithms are also chosen as comparison methods.

(1) The deep belief network (DBN) is one of the representatives of deep neural networks. Therefore, we choose a convolutional deep belief network (CDBN) as a comparison method [40].

(2) CNNs have excellent feature extraction capabilities. To improve the performance of CNNs, ResNet is chosen [41].

(3) The stacked autoencoder (SAE) is also widely used in fault diagnosis research. To meet the requirements of the model, the input 2D image is flattened into a 1D signal [42].

To verify the effect of PGD-SSTFT, the results of other commonly used signal processing methods are input into the model. These methods are the same as in Section 3.1. For comparison, the parameter settings of these comparison methods are also obtained after cross-validation.

The mean average precision (mAP), diagnosis accuracy and standard deviation are chosen as the indicators to evaluate model performance. mAP can be defined as

(24) $$\begin{array}{c} \begin{array}{c} Precision=\frac{TruePositives}{TruePositives+FalsePositives} \end{array} \end{array}$$

(25) $$\begin{array}{c} \begin{array}{c} Recall=\frac{TruePositives}{TruePositives+FalseNegatives} \end{array} \end{array}$$

AP is the precision averaged across all recall values between 0 and 1, which is obtained by calculating the area under the curve (AUC) of the precision x recall curve. In this paper, the mAP of each class is used to compare different detectors.

**Figure 9:**
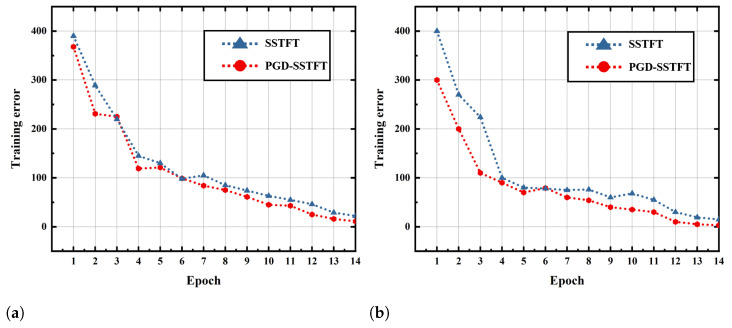
The loss curves of different methods. (**a**) Proposed method. (**b**) Yolo.

After the collected signal is processed and input to the model, training and testing are performed and the training errors are shown in Figure 9. The experiment is repeated ten times and the average results of the experiments are shown in the table. As Table 5 shows, the fault identification method proposed in this paper outperforms the Yolo model. The proposed method achieved the best mAP values, proving that this method is more effective in completing fault identification. In particular, when the PGD-SSTFT is used as input, the average diagnostic accuracy is 98.35%, which is approximately 3% higher than that of the Yolo model, proving that the method has better diagnostic performance.

Table 6 compares the results of the proposed method and those of other advanced fault diagnosis methods. The table shows that different fault diagnosis methods have achieved better diagnosis results after signal preprocessing; this finding indicates the necessity of applying signal processing technology in fault diagnosis. In addition, the results show that the proposed method has higher accuracy and stability takes less computing time than other methods. Overall, there is still room for improvement in terms of running time for intelligent algorithms. It is believed that this defect will be overcome in the future with continuous improvements in computer hardware.

**Figure 10:**
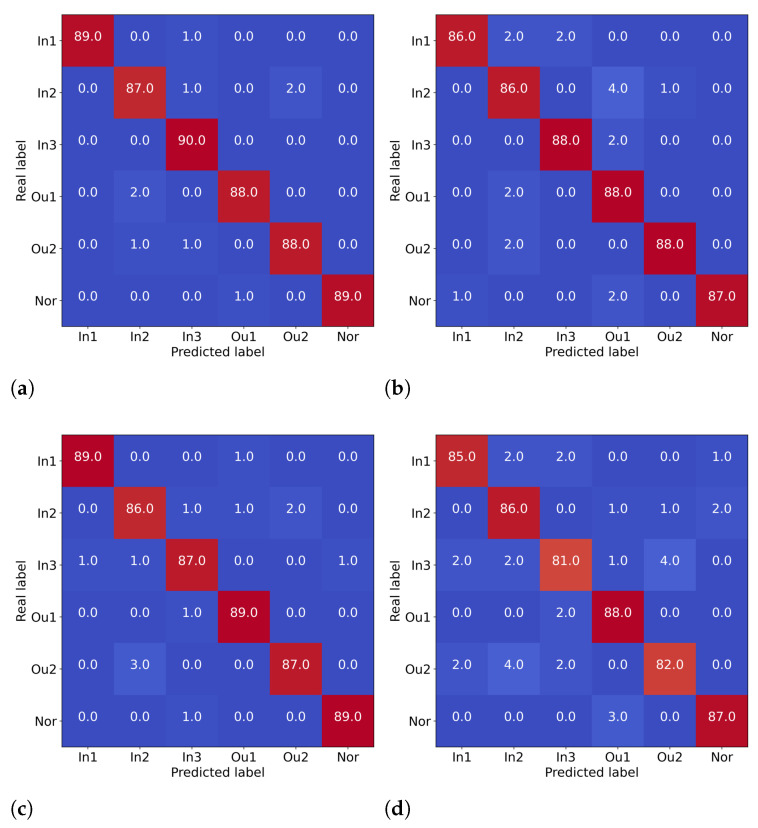
The confusion matrix for the testing set. (**a**) Proposed method. (**b**) CDBN. (**c**) ResNet. (**d**) SAE.

Figure 10 shows the confusion matrix of the fourth experimental diagnosis results of the proposed method and other methods. The rows represent the real labels for the different bearing status categories and the columns represent the predicted labels. The figure shows that the misclassified samples of different methods are concentrated mainly in the weak faults in the inner race and outer race, thus indicating that there are still some challenges in the weak fault diagnosis. However, it is still obvious that compared with the other methods, the proposed method shows higher diagnostic accuracy in different fault categories.

**Figure 11:**
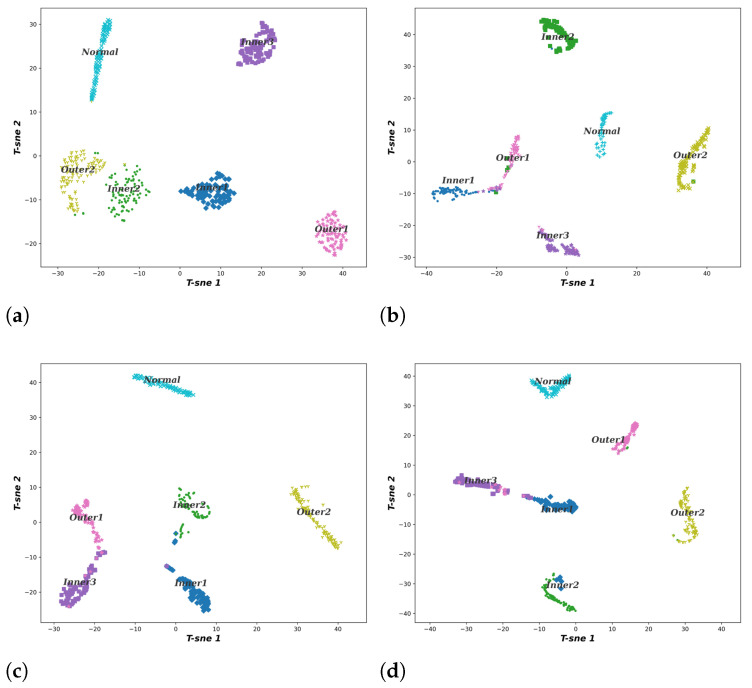
The T-SNE visualization result. (**a**) Proposed method. (**b**) CDBN. (**c**) ResNet. (**d**) SAE.

To better evaluate the learning ability of the proposed method, the T-SNE algorithm is utilized to visualize the high-dimensional features in the last fully connected layer of the different methods. The two-dimensional visualization results are shown in Figure 11, which demonstrates that the proposed method is more effective than other methods in feature identification of each state of the bearing and the feature distribution in each category is more concentrated. For other methods, there is an obvious overlap between the inner race and outer race because the model does not completely or incorrectly remove redundant information while learning features. This can easily lead to incorrect fault identification.

**Figure 12:**
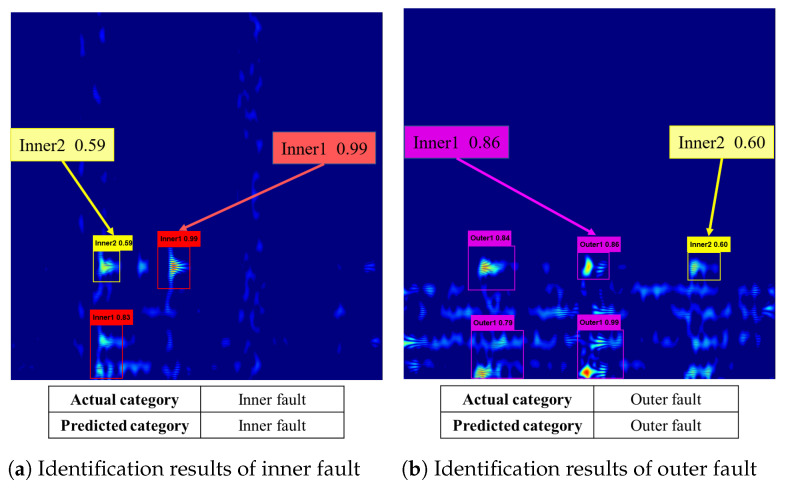
The fault identification results of the proposed method.

Figure 12 presents the final output of the marked results of the proposed method. According to the figure, both types are correctly predicted and the predicted probabilities of different fault types are marked, but there are still some false detections. However, the probability of a correct prediction still exceeds that of an incorrect prediction, so the model will output the correct fault type in the final fault identification.

## 6. Concluding Remarks

In this paper, a fault diagnosis strategy for rolling bearings based on sparse time-frequency analysis and Faster RCNN is proposed. To overcome the shortcomings of traditional time-frequency analysis methods, such as low resolution and cross-term interference, the SSTFT is employed to process the original signal. The results prove that the proposed method can effectively obtain high-resolution TFRs from bearing vibration signals and outperforms the classical nonsparse decomposition approaches. A fault diagnosis framework is built based on Faster RCNN. Multiple RoIs are used to determine the fault type of a sample, thus improving the accuracy and stability of diagnosis against traditional methods.

To verify the effectiveness of the proposed method, a benchmark bearing fault dataset is employed to identify different operating states of the bearing. The results indicate that this method has higher accuracy than traditional methods, can reliably identify different bearing faults and can accurately mark the position of the fault in the TFR. In addition, the effectiveness of the sparse time-frequency analysis is also validated by the experimental results. In view of the excellent performance of the proposed method, the focus of our future research will be fault diagnosis for compound faults.

## Figures and Tables

**Table 1:** Simulation parameters.

$f_{n}$	$T_{\mathrm{inner}}$	γ	$f_{r}$	$A_{0}$
7000 Hz	0.01	300	130 Hz	7.5

**Table 2:** Test bench parameters.

Parameter Description	Value
Bearing type	6203
Rotational speed	1500 rpm
Load torque	0.7 Nm
Radial force	1000 N
Sampling time	4 s
Sampling rate	64 kHz

**Table 3:** The rolling bearing operation conditions.

Data Index	Damage Method	Component	Fault Diameter (mm)	Characteristic of Damage *	Size of Training/Testing Samples
Inner1	Fatigue: Pitting	Inner race	6	A	180/50
Inner2	Fatigue: Pitting	Inner race	1	B	180/50
Inner3	Fatigue: Pitting	Inner race	2.5	A	180/50
Outer1	Plastic deform: Indentations	Outer race	<1	A	180/50
Outer2	Fatigue. Pitting	Outer race	2&3	B	180/50
Normal	—	—		—	180/50

* A: single point without repetitive damage. B: single point damage with random distribution.

**Table 4:** Model selection and influence.

Hyperparameter							Average Accuracy	Computer Time
The size of widow function	20						97.22%	-
	40						97.43%	-
	60						98.45%	-
	80						98.36%	-
	100						97.61%	-
The bound of error		1					93.91%	1141.0
		0.1					95.71%	1209.5
		0.01					97.82%	1395.8
Batch size			4				97.41%	1473.2
			8				98.09%	1330.1
			16				98.04%	1349.8
The base anchor size				8			98.82%	1339.5
				16			97.70%	1205.9
				32			96.59%	1101.6
Learning rate					0.1		94.23%	1305.6
					0.01		96.42%	1454.8
Dropout						0.25	97.56%	-
						0.5	95.44%	-
Base	60	0.01	8	8	0.01	0.25	98.35%	1342.9

**Table 5:** The test performance of different object detection methods.

Approach	Input	mAP	Diagnosis Accuracy	Standard Deviation
Proposed method	PGD-SSTFT	89.65%	98.35%	0.34
	SSTFT	88.43%	96.64%	0.33
	CWT	88.94%	97.15%	0.41
	WVD	79.55%	91.46%	0.67
Yolo	PGD-SSTFT	87.61%	95.27%	0.54
	SSTFT	86.54%	93.80%	0.51
	CWT	87.01%	94.94%	0.78
	WVD	80.94%	91.66%	0.84

**Table 6:** The test performance of all comparison methods.

Approach	Input	Diagnosis Accuracy	Standard Deviation	Computer Time
Proposed method	PGD-SSTFT	98.35%	0.34	1342.9
	SSTFT	96.64%	0.33	1457.5
	CWT	97.15%	0.41	1375.4
	WVD	91.46%	0.67	1307.2
CDBN	PGD-SSTFT	96.84%	0.45	1304.4
	SSTFT	96.77%	0.49	1423.9
	CWT	95.49%	0.45	1407.5
	WVD	90.81%	0.51	1464.9
ResNet	PGD-SSTFT	97.62%	0.22	1305.9
	SSTFT	96.24%	0.3	1433.9
	CWT	96.01%	0.21	1320.3
	WVD	93.17%	0.21	1322.5
SAE	PGD-SSTFT	94.57%	1.13	1290.5
	SSTFT	94.41%	1.09	1324.9
	CWT	93.29%	1.01	1318.3
	WVD	90.16%	0.3	1309.4

## Data Availability

Not applicable.
